# Supplementary material for: An integrated data framework for policy guidance during the coronavirus pandemic: Towards real-time decision support for economic policymakers
Source: PLoS One. 2022 Feb 14;17(2):e0263898. doi: 10.1371/journal.pone.0263898 (PMC8843231; doi:10.1371/journal.pone.0263898)
Supplement: S1 Table — Searches were conducted case insensitive. Spaces in the search terms were treated as wildcards where any two characters instead of the space also led to a match. In this way, we allowed a greater degree of variation in the search for Corona references. (PDF) [file pone.0263898.s001.pdf]

| | |
|---|---|
| Search terms (translated) | corona, corona virus, corona pandemic, corona crisis, covid 19, sars cov 2, wuhan virus, pandemic, 2019 ncov |
