# Supplementary material for: An integrated data framework for policy guidance during the coronavirus pandemic: Towards real-time decision support for economic policymakers
Source: PLoS One. 2022 Feb 14;17(2):e0263898. doi: 10.1371/journal.pone.0263898 (PMC8843231; doi:10.1371/journal.pone.0263898)
Supplement: S2 Table — Table shows the fraction (in %) of companies within the presented sector-size strata where we could find COVID-19 references on the corporate website in at least one of our web queries. Fractions reveal that larger firms are more likely to report about the virus on their websites. The numbers also show great heterogeneity across sectors. The last column presents the sample size of corporate website addresses across sectors. (PDF) [file pone.0263898.s002.pdf]

Sector	Size of company				
	Large	Medium	Small	Micro	Unknown
Business-related services	53.1	38.2	25.0	12.0	8.8
Wholesale & retail trade	38.4	30.3	24.1	14.4	12.8
Manufacturing	43.1	24.9	9.7	5.0	5.3
Health & social services	82.5	59.4	42.1	25.2	24.1
Accommodation & catering	62.1	40.1	29.1	18.8	15.0
Others	89.1	75.5	62.2	32.0	27.1
Creative industry & entertainment	66.7	66.1	56.3	37.6	30.7
Insurance & banking	75.6	60.0	38.4	33.4	38.5
Logistics & transport	60.9	32.0	13.3	8.0	7.4
Mechanical engineering	45.9	24.8	10.1	5.3	5.5
Food production	20.7	18.6	13.6	10.9	9.8
Chemicals & pharmaceuticals	40.1	23.7	10.7	6.6	6.2
Manufacturing of data processing equipment	53.2	35.6	17.5	7.4	5.9
Total	59.5	38.8	23.8	14.3	15.2
