# Supplementary material for: An integrated data framework for policy guidance during the coronavirus pandemic: Towards real-time decision support for economic policymakers
Source: PLoS One. 2022 Feb 14;17(2):e0263898. doi: 10.1371/journal.pone.0263898 (PMC8843231; doi:10.1371/journal.pone.0263898)
Supplement: S3 Table — Table shows the translation of EU’s NACE Revision 2 divisions [47] into the sector groupings used in this study. (PDF) [file pone.0263898.s003.pdf]

**Sectors**	**Divisions**
Business-related services	58-63, 68, 69-82
Manufacturing	5-9, 12-19, 23-25, 27, 31-33, 35-39, 41-43
Wholesale & retail trade	45-47
Health & social services	86-88, 94-96
Insurance & banking	64-66
Accommodation & catering	55, 56
Logistics & transport	49-53
Creative industry & entertainment	90-93
Mechanical engineering	28-30
Food production	10, 11
Chemicals & pharmaceuticals	20-22
Manufacturing of data processing equipment	26
Others	any division not listed above
