# Supplementary material for: An integrated data framework for policy guidance during the coronavirus pandemic: Towards real-time decision support for economic policymakers
Source: PLoS One. 2022 Feb 14;17(2):e0263898. doi: 10.1371/journal.pone.0263898 (PMC8843231; doi:10.1371/journal.pone.0263898)
Supplement: S4 Table — Table shows translation of firm characteristics into company size classes as defined by [54] and also used in this study. (PDF) [file pone.0263898.s004.pdf]

	Size of company			
	Micro	Small	Medium	Large
Number of employees	≤ 10	$11 - 49$	$50 - 249$	≥ 250
Annual turnover (in M €)	≤ 2	$2 - 10$	$10 - 50$	> 50
Annual balance sheet total (in M €)	≤ 2	$2 - 10$	$10 - 43$	> 43
