# Supplementary material for: An integrated data framework for policy guidance during the coronavirus pandemic: Towards real-time decision support for economic policymakers
Source: PLoS One. 2022 Feb 14;17(2):e0263898. doi: 10.1371/journal.pone.0263898 (PMC8843231; doi:10.1371/journal.pone.0263898)
Supplement: S5 Table — Table shows three website text examples for each of the five context classes retrieved from distinct corporate websites. (PDF) [file pone.0263898.s005.pdf]

Categories	Description	Examples (translated)
Problem	Firm reports about adverse impacts of the pandemic on its business operations.	Due to the Corona pandemic, [redacted] & [redacted] are closed.<br>[redacted] has been cancelled due to the increasing concerns and escalated circumstances surrounding the recent coronavirus (COVID-19) outbreak.<br>The Corona pandemic is not only affecting ongoing [redacted] projects, but also the current selection rounds of the 13th and 14th funding seasons.
No problem	Firm indicates that the pandemic has no negative impacts on its business operations.	We are there for you 24/7 as usual despite Corona!<br>Your [redacted] advisor stands by your side - also in times of COVID-19.<br>Corona - we are your stable partner, even in difficult times.
Adaption	Firm reports that it is adapting to the new economic circumstances.	We have also upgraded our IT and telecommunications system. Our employees are now also able to ensure that you are looked after from home, should this be necessary. Since we receive new information on the development of the coronavirus, the measures and the safety precautions every day, we will continue to monitor the development and react to it.<br>Within our emergency opening times, we particularly take care of those who are currently performing at their best for our society in view of the coronavirus crisis and who depend on their glasses for their work.<br>We have therefore decided to adapt our services to the current situation and to limit them until further notice. Although we want to continue to provide you with all indispensable services, we also want to meet the recommendations of the federal government on how to deal with the corona virus.
Information	Firm reports generally, not necessarily in a business-context, about the pandemic.	The corona pandemic affects each of us now and in the near future. There are many uncertainties and resulting (insurance) issues. What about entitlement to holiday cancellations, health protection abroad and coverage in the event of business interruption are just a few of the questions.<br>In cooperation with the software provider [redacted], the Bundesverband Pflegemanagement (Federal Association of Care Management) is launching a platform to recruit former care professionals to cope with the currently dramatic challenges facing care against the background of the Corona crisis.<br>The Association of Statutory Health Insurances has signaled support for companies that are in acute liquidity difficulties due to the Corona crisis. In particular, the interest-free deferral of contributions is massively facilitated.
Unclear	COVID-19 reference does not come with further clearly distinguishable content.	Current situation COVID-19.<br>COVID-19 and how it affects us.<br>Together against Corona.
