# Supplementary material for: An integrated data framework for policy guidance during the coronavirus pandemic: Towards real-time decision support for economic policymakers
Source: PLoS One. 2022 Feb 14;17(2):e0263898. doi: 10.1371/journal.pone.0263898 (PMC8843231; doi:10.1371/journal.pone.0263898)
Supplement: S6 Table — Table shows sector level impact values as displayed in Fig 3. Impact values are defined as the proportion of companies that communicated about the pandemic in the respective context within that sector. The unweighted average of the impact values across all sectors forms the grey-shaded reference area in Fig 3. (PDF) [file pone.0263898.s006.pdf]

**Sector**	**problem**	**no problem**	**adaption**	**infor-mation**	**unclear**
Accommodation & catering	0.5802	0.0308	0.4563	0.0857	0.3244
Business-related services	0.2368	0.0648	0.6762	0.3829	0.5400
Chemicals & pharmaceuticals	0.1782	0.1079	0.6248	0.2359	0.5922
Creative industry & entertainment	0.7747	0.0055	0.3451	0.2705	0.4123
Food production	0.3516	0.0906	0.5863	0.1077	0.3937
Health & social services	0.3615	0.0346	0.6957	0.3469	0.4724
Insurance & banking	0.1289	0.2118	0.8654	0.3662	0.5523
Logistics & transport	0.2940	0.0893	0.6780	0.2763	0.5130
Manufacturing	0.2753	0.0884	0.6207	0.2049	0.4206
Manufacturing of data processing equipment	0.1930	0.1329	0.6727	0.1944	0.5860
Mechanical engineering	0.1982	0.1357	0.6736	0.1724	0.5246
Others	0.5599	0.0138	0.5452	0.4073	0.5450
Wholesale & retail trade	0.2642	0.0877	0.6190	0.2455	0.4879
Average (unweighted)	0.3382	0.0842	0.6199	0.2536	0.4896
