# Supplementary material for: An integrated data framework for policy guidance during the coronavirus pandemic: Towards real-time decision support for economic policymakers
Source: PLoS One. 2022 Feb 14;17(2):e0263898. doi: 10.1371/journal.pone.0263898 (PMC8843231; doi:10.1371/journal.pone.0263898)
Supplement: S7 Table — Table shows impact values of wholesale companies disaggregated by NACE Revision 2 classes (4-digit-level) [47]. Impact values are defined as the proportion of companies that communicated about pandemic-related problems within the respective subsector. N refers to the number of observations in the subsector. (PDF) [file pone.0263898.s007.pdf]

Sector subgroup	NACE Rev. 2 class	impact value problem class	N
Wholesale of			
- machinery for the textile industry and of sewing and knitting machines	4664	0.4286	7
- live animals	4623	0.3929	28
- flowers and plants	4622	0.3729	59
- other intermediate products	4676	0.3548	31
- waste and scrap	4677	0.3492	63
- watches and jewelry	4648	0.3478	46
- textiles	4641	0.3444	90
- beverages	4634	0.3189	185
- machine tools	4662	0.3061	49
- perfume and cosmetics	4645	0.2903	62
- agricultural machinery, equipment and supplies	4661	0.2878	205
- clothing and footwear	4642	0.2791	129
- grain, unmanufactured tobacco, seeds and animal feeds	4621	0.2593	108
- other household goods	4649	0.2575	435
- furniture, carpets and lighting equipment	4647	0.2568	148
- fruit and vegetables	4631	0.2558	43
- wood, construction materials and sanitary equipment	4673	0.2405	499
- metals and metal ores	4672	0.2342	111
- mining, construction and civil engineering machinery	4663	0.2308	26
- electronic and telecommunications equipment and parts	4652	0.2143	84
- coffee, tea, cocoa and spices	4637	0.2105	38
- hardware, plumbing and heating equipment and supplies	4674	0.2088	340
- electrical household appliances	4643	0.1861	317
- other machinery and equipment	4669	0.1854	712
- office furniture	4665	0.1842	38
- chemical products	4675	0.1835	158
- other office machinery and equipment	4666	0.1774	62
- other food, including fish, crustaceans and molluscs	4638	0.1729	133
- dairy products, eggs and edible oils and fats	4633	0.1538	13
- china and glassware and cleaning materials	4644	0.1528	72
- pharmaceutical goods	4646	0.1406	448
- computers, computer peripheral equipment and software	4651	0.1331	248
- solid, liquid and gaseous fuels and related products	4671	0.1301	123
- food, beverages and tobacco (non-specialized)	4639	0.1132	53
- tobacco products	4635	0.1000	10
- sugar and chocolate and sugar confectionery	4636	0.1000	10
- meat and meat products	4632	0.0482	83
