# Supplementary figures and images for: An integrated data framework for policy guidance during the coronavirus pandemic: Towards real-time decision support for economic policymakers
Source: PLoS One. 2022 Feb 14;17(2):e0263898. doi: 10.1371/journal.pone.0263898 (PMC8843231; doi:10.1371/journal.pone.0263898)

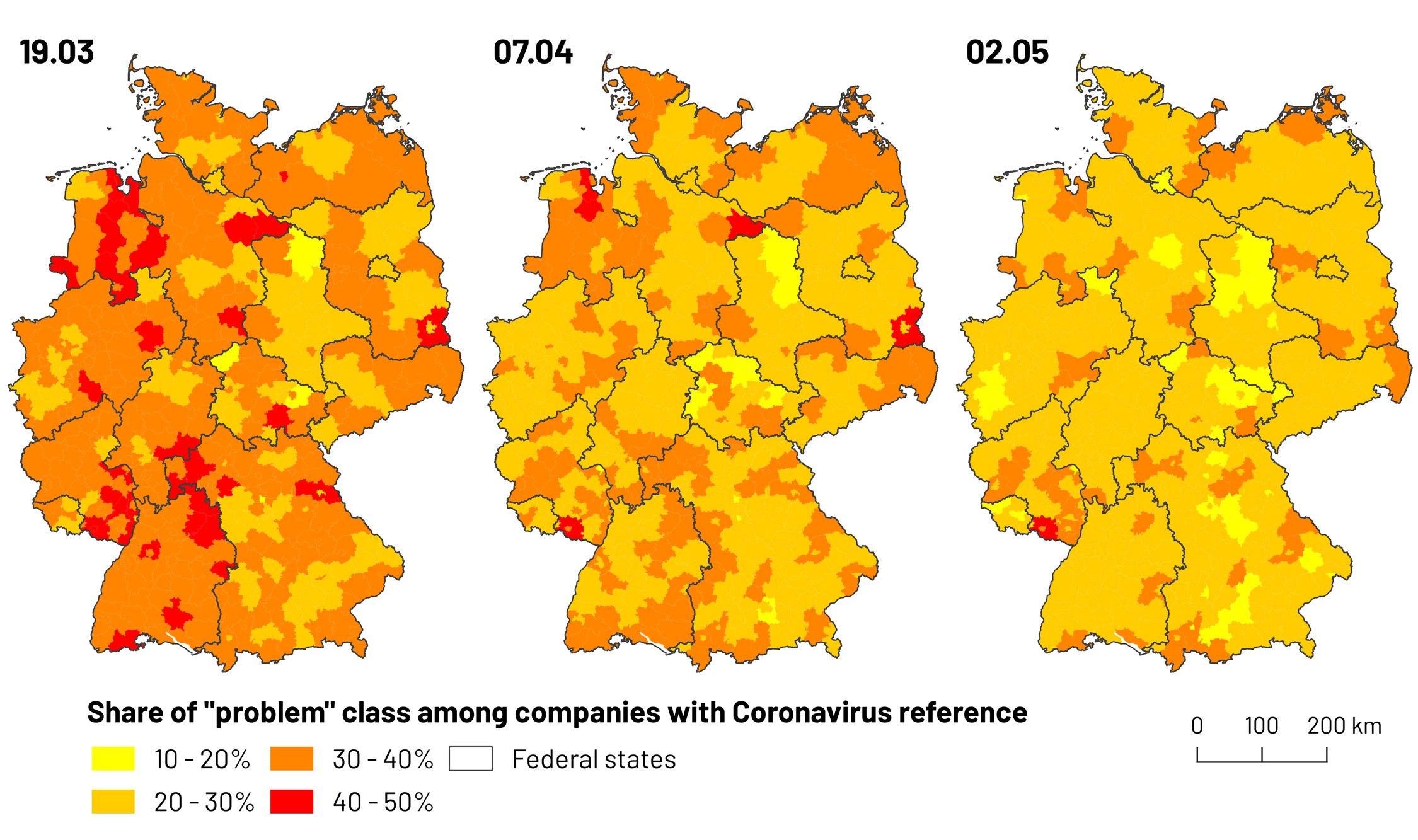

Supplement: S1 Fig — Figure shows regional impact values to demonstrate the presented framework’s capability to monitor regional hotspots where comparatively many companies are negatively affected by the shock. Impact values are presented for three selected web queries in March, April and May 2020. Regional impact values show at the beginning of the pandemic strong problem reporting of companies located in cross-border regions. Investigation of the text references showed that these values were driven by specialized companies located at transportation hubs that were virtually unused during the lockdown. Towards the end of the first economic shutdown beginning of May, problem reports diminished. Impact values are defined as the proportion of companies that reported about pandemic-related problems within that region. (TIF) [file pone.0263898.s008.tif]
