# Supplementary material for: An integrated data framework for policy guidance during the coronavirus pandemic: Towards real-time decision support for economic policymakers
Source: PLoS One. 2022 Feb 14;17(2):e0263898. doi: 10.1371/journal.pone.0263898 (PMC8843231; doi:10.1371/journal.pone.0263898)
Supplement: S1 Appendix — The business surveys presented in this study are the result of a joint research project between the polling agency KANTAR and ZEW—Leibniz Centre for European Economic Research funded by the German Federal Ministry for Economic Affairs and Energy (BMWi). Scope of the project was to provide early insights on the effects of the pandemic on the German business sector based on website analyses and survey data. The sampling strategy followed a stratified random sample drawn from the MUP which comprises the near universe of active firms in Germany [37]. Stratification was conducted by industry (see S3 Table) and employee size classes (see S4 Table) and ensured sufficient regional coverage across federal states. Computer aided telephone interviews following a predefined stratification matrix by size classes and industries were conducted. The predefined stratification matrix formed the basis for gross sampling as well as for sampling management during fieldwork. A target of at least N = 30 interviews in the industries ensured that sufficient observations were available at the sector-level to ensure credible conclusions for all sectors as it is done in this study. Overall, three recurring survey waves over the period from April to September 2020 have been conducted. Not all firms agreed upon processing their responses beyond the scope of the aforementioned research project. These firms are excluded from the analyses in this paper. The table below provides further details concerning the total number of interviewed companies (Noverall) and the number of firms included in this study (N) for each of the survey waves. As far as possible, companies were continuously surveyed in all three survey waves. If companies refused to participate again or could not be reached in a subsequent wave, new companies were drawn from the stratified gross sample in order to meet the target observation number of the respective survey wave. (PDF) [file pone.0263898.s009.pdf]

**Wave**	**Field period**	$N_{overall}$	N
1	14-Apr-20 – 23-Apr-20	500	469
2	8-Jun-20 – 23-Jun-20	1,000	958
3	24-Sep-20 – 13-Oct-20	1,000	966
