# Supplementary material for: An integrated data framework for policy guidance during the coronavirus pandemic: Towards real-time decision support for economic policymakers
Source: PLoS One. 2022 Feb 14;17(2):e0263898. doi: 10.1371/journal.pone.0263898 (PMC8843231; doi:10.1371/journal.pone.0263898)
Supplement: Attachment — Submitted filename: Response_to_Reviewers.pdf [file pone.0263898.s010.pdf]

Response to Reviewer #1

General Comment: This work introduces an integrated data framework for guiding policymakers during a crisis in a timely and cost-effective manner. The core of this work focuses on finding different strategies to analyze various data sources from companies that can provide the bigger picture during the Covid-19 pandemic, whether it's in an early stage near real-time, in a follow-up, or a retrospective stage (in the aftermath of the shock). The authors conclude that their framework provides policymakers with guidance for their economic support measures in times of sudden shocks.
Response: *Thank you very much for thoroughly reading our paper and identifying the main contributions. We hope that by carefully considering all of your comments/suggestions, we were able to further improve our paper and provide a more specific argument for the usefulness of the proposed framework.*

General Comment: Strengths
a) This work provides an interesting study of the economic sector struggles during Covid-19.
b) The idea to combine data from different data sources (e.g., online, surveys, official data) to aid in forecasting is very good, although not new.
c) Most of the references are new and relevant.
Response: *Thank you very much for highlighting the strengths of the paper.*

Comment 1 a): Title of the paper
The title is more generic than what the article presents. The authors should deliver a "data framework for policy guidance in times of dynamic economic shocks" but the solution is very specific to the Covid-19 scenario.
Response: *We agree that the presented near real-time assessment to assist policymakers with timely information at an early stage of an economic shock is specific to the coronavirus pandemic. For this reason, we have adjusted the title to 'An integrated data framework for policy guidance during the coronavirus pandemic: Towards real-time decision support for economic policymakers'. We hope that this makes the title less generic but gives a more specific idea about the main contributions of the paper: Assisting policymakers with timely information and empirical evidence throughout all stages of the pandemic to design more targeted short-term measures in the first place, but also to continuously update political decision-makers' information basis for effective response measures in the medium-term. Nonetheless, we argue that the idea to integrate timely online sources with more traditional but less timely policy data generalizes to other economic shocks. Integrating these data sources, as proposed in the paper, allows policymakers not only to react more swiftly and targeted but also allows to design medium to long-term stimulus packages based on a rich set of information that has been continuously updated over all stages of a shock. We provide further details on how our proposed approach generalizes to other types of shocks and which extension are required to provide a more automated and universally applicable data framework both in our response to Comment 3 and in the revised version of the paper.*

Comment 1 b): Manual steps within the framework
It will be very hard to generalize (to other types of crisis) as most of the steps are done manually and not automatically, e.g., they somehow chose some specific keywords/terms to search in the text (section 3.1) and some classes for the classifier. Something like automated detection of the keywords would make the solution more general.
Response: *This is a valid point that we had not previously addressed in the paper. As written above, we have decided to adjust the title of the paper based on your comments. It should now be clearer that we are not providing a ready-to-use framework for all kinds of economic shocks, but rather an initial proposal with an explicit use case (the coronavirus pandemic). That said, optimally, in the future (and after further research and development), we should aim for a framework that is at least mostly automated and can be universally deployed. This includes the points you mentioned about automated keyword and class generation. To address this issue, we have added section 3.4 in the paper where we show possible development paths towards such a system. Thank you for nudging us in this direction.*

Comment 1 c): Evaluation of the classification model
Even the evaluation of the model (XLM-RoBERTa) was done only manually, instead of also using specific evaluation metrics. They did not discuss the precision, accuracy, or recall of the model.
Response: *We agree that a detailed discussion of the performance our classification model along with a presentation of the respective performance metrics is an important aspect that was missing in the previous version of the paper. As part of the revision process, we have thus undertaken a thorough performance evaluation of the transformer-based language model that we have fine-tuned for the task of classifying in which context the companies in our sample communicated about the coronavirus pandemic. For this purpose, we have created an out-of-sample test set (consisting of 1,000 firm communications that we did not use for fine-tuning the language model) for which we engaged two independent annotators to manually assign each of the test references to one of the five context categories. In this way, we could not only evaluate the classifier's performance based on the suggested metrics, but we could also discuss how distinguishable our previously introduced context classes were given an analysis of the inter-annotator agreement. A detailed discussion of the classifier's performance, along with the presentation of the underlying metrics, can be found in Table 2 and in the text above in the revised version of the paper.*

Comment 2: Theoretical and experimental analyses

The theoretical and experimental analyses of the paper are not rigorous.

Response: *We agree that the theoretical and experimental analyses of the paper can be improved upon. Thus, we added a more detailed description of the transformer-based classification model in the first place. Specifically, we improved the intuition about the transfer-learning approach that we used to classify the text passages, described how we handled unbalanced data during fine-tuning the model and added specific insights to the XLM-RoBERTa model. You'll find these extensions in Table 1 and in the text above in the revised version of the paper.*

Comment 3 a): Generalizability to other types of shocks

By only analyzing the Covid-19 scenario, they do not demonstrate that their solution is efficient and optimal in a general crisis situation.

Response: *You are right that the first stage of our proposed framework, i.e. tracking the early effects after an economic shock via company communication patterns, is specific to the coronavirus pandemic. However, the idea to integrate timely online sources with more traditional but less timely policy data can be generalized to other types of economic shocks and provides an important step towards a more timely and holistic approach to policy guidance. In this respect, the coronavirus pandemic has shown that the current approach to assist political decision-makers with information is incomplete. This has also been acknowledged by a recent issue of The Economist (Oct 23rd 2021 edition titled 'Instant economics: The real-time revolution') which states that 'without the time to wait for official surveys to reveal the effects of the virus or lockdowns' (The Economist(1), 2021, para. 5) there was no alternative for policymakers but to 'operate[...] in a fog' (The Economist(2), 2021, para. 11). Especially in the early stage of the Corona pandemic, empirical impact data was missing to provide decision support for economic policymakers. Despite their lack of timeliness, surveys and official statistics are nonetheless important instruments for designing medium-term to long-term responses. We argue that also in times of economic shocks they will continue to provide empirical justification for the design of policy measures, but more work should be dedicated in researching how more timely data from online sources can complement these traditional forms of policy data, especially if empirical evidence is required for immediate government responses. Such efforts pose an important step towards a policy framework that allows to continuously update political decision makers in times of rapid change.*

Besides, the near real-time assessment of the pandemic via corporate communication patterns (and the leading indication of the generated indicators), the second contribution of the paper is thus to move towards a strategy that complements various crisis-related data sources for more informed policy-making. It is the idea that timely insights from online sources may not only serve as leading indicators but are equally important to design more targeted surveys which in turn reveal more detailed information how firms are affected by the shock. Integrating these data sources, as proposed in the paper, allows policymakers not only to react more swiftly and targeted but also allows to design medium to long-term stimulus packages based on a rich set of information that has been continuously updated over all stages of the shock. We argue that this holistic approach bears the potential to be applicable to a wider range of economic shocks, but clearly requires a higher degree of automation for more general crisis situations. In section 3.4 of the revised paper, we have therefore added a paragraph where we outline this broader view on how to integrate various data sources for policy guidance and how our framework could be leveraged to a more automated and universal level. Also we have made in-text adjustments at the beginning of section 3.2 to further sharpen the integrative nature of our framework. In this vein, we also see our study as a contribution to the recent literature that has investigated the value of combining small data sources such as surveys with large scale online sources. In the social sciences, the integration of such heterogeneous sources of information is considered to be high and has recently been documented in various studies (e.g. Callegaro et al. (2018), Conrad et al. (2019), Eck et al. (2021), Baghal et al. (2021)). In this context, our paper contributes to a specific use case where the combination of small and big data sources allows overcoming information deficits to reduce the risk of policy errors. We hope that with the above explanation, as well as with the adjustments and extensions in the revised version of the paper, we could underline our efforts to move towards a more timely and holistic data framework for policy guidance that, given further development, bears the potential to be more generally applicable.

Comment 3 b): In-depth insights of the study

Also, at the start of the lockdown was very clear what sectors are going to suffer the most. I am not sure what in-depth insights this study offers.

Response: *You are right that an educated guess could be made at the beginning of the pandemic regarding the sector-specific impact of the containment measures. However, we argue that in terms of accountability there is an important difference between an educated guess and empirical evidence. As pointed out in our study, the coronavirus pandemic has led to an increased demand of evidence-based decision support among political decision-makers to legitimize policy action (Weible et al., 2020). The empirical evidence provided by the real-time impact assessment in our study is an important step towards evidence-based decision making in situations where other information sources fail to deliver timely empirical evidence.*

Moreover, to better demonstrate the insights of our study, we show how our approach allows to study impact variation among sectoral subgroups. Unlike business surveys, the large sample size of the webdata-generated impact values makes such a fine granular assessment possible. More precisely, in the revised version of the paper, we analyze variation in the communication patterns of wholesale companies active in close to 40 different product markets (see

the extension below Fig 3 and S7 Table in the revised paper). We argue that such a fine granular effect differentiation based on firms specialized in the supply of different goods and commodities has the potential to indicate the risk of supply shortages of specific products early on.
Another example of heterogeneity at the sector level is companies with local supply relationships on the one hand and companies that rely on international supply chains or even specialize in cross-border logistics of goods and products on the other hand. The supply chains, which are (still to this day) heavily impacted, then have strongly idiosyncratic effects on the individual firms, which can be mapped by the method we have presented. These individual effects then also influence the geographic pattern of the impact of the lockdown measures, as the map we have added to the paper suggests (see S8 Figure).

Comment 4: Research contribution
To sum up, the primary issue with this paper is that the contribution as a piece of research is not clear.
Response: *The core contribution of this paper is an experimental analysis to overcome information deficits political decision makers have been facing in a situation were swift response was required. In this context, the coronavirus pandemic has highlighted the weaknesses of survey-based policy data which cannot be made available without significant time lags. For swift and targeted policy responses, they do not only lack timeliness but also do not serve well to study variation across geographies and firm-specific subgroups given their small scale nature. In light of these deficiencies, it is an open research question how to derive early and policy-relevant insights from less traditional sources, typically unstructured online sources that are generated at much higher frequency and in real-time. Economic research, as important guide to public-sector decision-making, can contribute to overcome these information deficits. For the case of the coronavirus pandemic, we show that indicators from corporate communication patterns, as they could be extracted from a large sample of corporate websites, revealed sectoral and regional variation of the pandemic at near real-time. They also served as leading indicators for liquidity shortfalls at firm-level. We see these early findings as an empirical guide for policy actors to initiate more targeted policies, in a situation where other data cannot provide information on the underlying dynamics.*
Moreover, as also discussed in the responses above, we aim for a more timely and holistic data approach by integrating different sources of policy-relevant data once they become available over the course of an economic shock. We hope that with this explanation we could further clarify the research contribution of this study. To this aim, we have also adjusted parts in the introduction and the conclusion to provide a more focused line of argumentation concerning the paper's research contribution.

General Comment: Figures and tables
The figures and some of the tables were not added in the article, they can be found at the end of the document (all the figures) or can be downloaded separately (tables Si_Table).
Response: *You are right that both figures and tables which are part of the appendix are not included in the text. We understand that the paper would be more convenient to read, if they were directly included. However, we follow PLOS ONE's Latex guideline which states that 'figures should be uploaded separately from your manuscript file' and that 'figures generated using LaTeX should be extracted and removed from the PDF before submission'. Since we have generated our figures using Latex, we were required to add these as separate files. We apologize for any inconvenience caused.*

Response to Reviewer #2

General Comment: The paper proposes a framework for guiding policymakers in the context of a major economic event by analyzing small and big data. The topic is interesting and worth investigating, given the recent COVID-19 economic shock. The paper is well structured, includes a comprehensive enough literature review and uses recent transformer-based language models. However, many aspects of the paper are described in rather vague terms.
Response: *Thank you very much for these encouraging words. We hope that with a detailed performance evaluation of the classification model and a more detailed discussion of the results, along with additional information about the survey, we have been able to further improve our paper.*

Comment 1: Detailed information and evaluation of the classification model
a) Additional details should be provided regarding the classifier used for categorizing the announcements on the companies' websites. The current description can be considered quite vague. For example, the paper should state how many of the 4,347 manually classified text passages belonged to each considered category. Has the dataset been a balanced or an unbalanced one?
Response: *We agree this is a relevant point to mention. To this aim, we have added Table 1 in the revised version of the paper which gives an overview of the class distribution in the training data. From this, one can see that the class distribution is rather unbalanced as especially the 'no problem' class is underrepresented with less than 6% in the training data. In the next response, we explain how we handled this imbalance in the training process.*

b) How have the authors handled the training process if the training dataset has been unbalanced?
Response: *In order to handle the unbalancedness of the training data, we have employed class weights inversely proportional to the respective class frequency in the training set. We have added a detailed explanation of this approach in the revised version of the paper (see the in-text extension above Table 1 in the revised version of the paper).*

c) Additionally, what happened when a disagreement between the two annotators has been encountered?
Response: *For the generation of the training sample, we did not conduct* ***co****-annotation, but split the 4,273 text passages across independent annotators to let them be annotated individually. At this stage of the project, we refrained from letting one and the same text passage be annotated from multiple annotators, but rather focused on a timely implementation of the webdata-based monitoring framework, for which a sufficiently large number of training samples was key. However, as part of the revision process, we have created an out-of-sample test set (consisting of 1,000 firm communications that we did not use for fine-tuning the language model) and engaged two independent annotators to manually assign each of the test references to one of the five context categories (contrary to the previous performance evaluation where we asked annotators to decide whether the model's classification on a test set has been right or wrong). In this way, we could not only evaluate the classifier's performance based on the suggested metrics, but we could also discuss how distinguishable our previously introduced context classes were given an analysis of the inter-annotator agreement. A detailed discussion of the classifier's performance, along with the presentation of the underlying metrics, can be found in Table 2 and in the text above in the revised version of the paper.*

d) The paper should evaluate the performance of the classifier used for using the standard metrics such as Precision, Recall, Accuracy and F1-Score.
Response: *We agree that this is a valid point that we had not previously addressed in the paper. For this reason, as already mentioned in the previous response, we have undertaken a thorough performance evaluation of the transformer-based language model that we have fine-tuned for the task of classifying in which context firms communicated about the coronavirus pandemic. Based on the co-annotated out-of-sample test set we could evaluate the classifier's performance in detail using the proposed metrics. Again, see Table 2 and the text above this table in the revised version of the paper for more details.*

e) Numerical values should also be provided in the text discussing Figure 3. The authors could also choose to mention the values in a table.
Response: *We agree that precise numerical values of the sector-specific impact values should be included in the paper. To this aim, we have added numerical values in the text in order to underpin the conclusions that we draw from Figure 3 (see in-text adjustments above Figure 3). We have also followed your suggestion and added a table that displays all the numerical impact values as supporting information (S6 Table).*

Comment 2: Detailed information on the survey
Precise information regarding the survey should also be included. The current version of the paper does not for example mention the exact number of companies that have completed the survey in the text of the paper (only included in Table 1).
Response: *Thank you for pointing this out. To be more precise on the conducted surveys, we have added detailed information concerning sampling strategy, field work and survey responses as supporting information under S7 Appendix. Also we have added the exact number of distinct companies that have been interviewed in the text of the paper.*
